# The regularized CQ algorithm without *a priori* knowledge of operator norm for solving the split feasibility problem

**DOI:** 10.1186/s13660-017-1480-2

**Published:** 2017-09-05

**Authors:** Ming Tian, Hui-Fang Zhang

**Affiliations:** 1 College of Science, Civil Aviation University of China, Tianjin, 300300 China; 2 Tianjin Key Laboratory for Advanced Signal Processing, Civil Aviation University of China, Tianjin, 300300 China

**Keywords:** 58E35, 47H09, 65J15, split feasibility problem, regularized CQ algorithm, minimum-norm solution, strong convergence, operator norm

## Abstract

The split feasibility problem (SFP) is finding a point $x\in C$ such that $Ax\in Q$, where *C* and *Q* are nonempty closed convex subsets of Hilbert spaces $H_{1}$ and $H_{2}$, and $A:H_{1}\rightarrow H_{2}$ is a bounded linear operator. Byrne’s CQ algorithm is an effective algorithm to solve the SFP, but it needs to compute $\|A\|$, and sometimes $\|A\| $ is difficult to work out. López introduced a choice of stepsize $\lambda_{n}$, $\lambda_{n}=\frac{\rho_{n}f(x_{n})}{\|\nabla f(x_{n})\| ^{2}}$, $0<\rho_{n}<4$. However, he only obtained weak convergence theorems. In order to overcome the drawbacks, in this paper, we first provide a regularized CQ algorithm without computing $\|A\|$ to find the minimum-norm solution of the SFP and then obtain a strong convergence theorem.

## Introduction

Let $H_{1}$ and $H_{2}$ be real Hilbert spaces and let *C* and *Q* be nonempty closed convex subsets of $H_{1}$ and $H_{2}$, and let $A:H_{1}\rightarrow H_{2}$ be a bounded linear operator. Let $\mathbb {N}$ and $\mathbb{R}$ denote the sets of positive integers and real numbers.

In 1994, Censor and Elfving [1] came up with the split feasibility problem (SFP) in finite-dimensional Hilbert spaces. In infinite-dimensional Hilbert spaces, it can be formulated as

1.1 $$ \textit{Find }x\in C\textit{ such that }Ax\in Q, $$

where *C* and *Q* are nonempty closed convex subsets of $H_{1}$ and $H_{2}$, and $A:H_{1}\rightarrow H_{2}$ is a bounded linear operator. Suppose that SFP (1.1) is solvable, and let *S* denote its solution set. The SFP is widely applied to signal processing, image reconstruction and biomedical engineering [2–4].

So far, some authors have studied SFP (1.1) [5–17]. Others have also found a lot of algorithms to study the split equality fixed point problem and the minimization problem [18–20]. Byrne’s CQ algorithm is an effective method to solve SFP (1.1). A sequence $\{x_{n}\}$, generated by the formula

1.2 $$ x_{n+1}=P_{C}\bigl(x_{n}-\lambda_{n}A^{*}(I-P_{Q})Ax_{n} \bigr), \quad\forall n\geq0, $$

where the parameters $\lambda_{n}\in(0,\frac{2}{\|A\|^{2}})$, $P_{C}: H\rightarrow C$, and $P_{Q}: H \rightarrow Q$, is a set of orthogonal projections.

As is well-known, Cencor and Elfving’s algorithm needs to compute $A^{-1}$, and Byrne’s CQ algorithm needs to compute $\|A\|$. However, they are difficult to calculate.

Consider the following convex minimization problem:

1.3 $$ \min_{x\in C}f(x), $$

where

1.4 $$\begin{aligned}& f(x)=\frac{1}{2}\big\| (I-P_{Q})Ax\big\| ^{2}, \end{aligned}$$

1.5 $$\begin{aligned}& \nabla f(x)=A^{*}(I-P_{Q})Ax, \end{aligned}$$

$f(x)$ is differentiable and the gradient ∇*f* is *L*-Lipschitz with $L>0$.

The gradient-projection algorithm [21] is the most effective method to solve (1.3). A sequence $\{x_{n}\}$ is generated by the recursive formula

1.6 $$ x_{n+1}=P_{C}(I-\lambda_{n} \nabla f)x_{n}, \quad\forall n\geq0, $$

where the parameter $\lambda_{n}\in(0,\frac{2}{L})$. Then we know that Byrne’s CQ algorithm is a special case of the gradient-projection algorithm.

In Byrne’s CQ algorithm, $\lambda_{n}$ depends on the operator norm $\| A\|$. However, it is difficult to compute. In 2005, Yang [22] considered $\lambda_{n}$ as follows:

$$ \lambda_{n}:=\frac{\rho_{n}}{\|\nabla f(x_{n})\|}, $$

where $\rho_{n}>0$ and satisfies

$$ \sum_{n=0}^{\infty}\rho_{n} = \infty,\qquad \sum_{n=0}^{\infty}\rho_{n}^{2} < \infty. $$

In 2012, López [23] introduced $\lambda_{n}$ as follows:

$$ \lambda_{n}:=\frac{\rho_{n}f(x_{n})}{\|\nabla f(x_{n})\|^{2}}, $$

where $0<\rho_{n}<4$. However, López’s algorithm only has weak convergence.

In 2013, Yao [24] introduced a self-adaptive method for the SFP and obtained a strong convergence theorem. However, the algorithm is difficult to work out.

In general, there are two types of algorithms to solve SFPs. One is the algorithm which depends on the norm of the operator. The other is the algorithm without *a priori* knowledge of the operator norm. The first type of algorithm needs to calculate $\|A\|$, but $\|A\|$ is not easy to work out. The second type of algorithm also has a drawback. It always has weak convergence. If we want to obtain strong convergence, we have to use the composited iterative method, but then the algorithm is difficult to calculate. In order to overcome the drawbacks, we propose a new regularized CQ algorithm without *a priori* knowledge of the operator norm to solve the SFP and we obtain a strong convergence theorem.

Consider the following regularized minimization problem:

1.7 $$ \min_{x\in C}f_{\beta}(x):=f(x)+\frac{\beta}{2}\|x \|^{2}, $$

where the regularization parameter $\beta>0$. A sequence $\{x_{n}\}$ is generated by the formula

1.8 $$ x_{n+1}=P_{C}\bigl(I-\lambda_{n}(\nabla f+ \beta_{n}I)\bigr)x_{n}, \quad\forall n\geq0, $$

where $\nabla f(x_{n})=A^{*}(I-P_{Q})Ax_{n}$, $0<\beta_{n}<1$, and $\lambda_{n}=\frac{\rho_{n}f(x_{n})}{\|\nabla f(x_{n})\|^{2}}$, $0<\rho _{n}<4$. Then, under suitable conditions, the sequence $\{x_{n}\}$ generated by (1.8) converges strongly to a point $z\in S$, where $z=P_{S}(0)$ is the minimum-norm solution of SFP (1.1).

## Preliminaries

In this part, we introduce some lemmas and some properties that are used in the rest of the paper. Throughout this paper, let $H_{1}$ and $H_{2}$ be real Hilbert spaces, $A:H_{1}\rightarrow H_{2}$ be a bounded linear operator and *I* be the identity operator on $H_{1}$ or $H_{2}$. If $f:H\rightarrow\mathbb{R}$ is a differentiable functional, then the gradient of *f* is denoted by ∇*f*. We use the sign ‘→’ to denote strong convergence and use the sign ‘⇀’ to denote weak convergence.

### Definition 2.1

See [25]

Let *D* be a nonempty subset of *H*, and let $T:D\rightarrow H$. Then *T* is firmly nonexpansive if

$$\|Tx-Ty\|^{2}+\big\| (I-T)x-(I-T)y\big\| ^{2}\leq\|x-y \|^{2}, \quad\forall x, y\in D. $$

### Lemma 2.2

See [26]

*Let* $T:H\rightarrow H$ *be an operator*. *Then the following are equivalent*:

(i) *T* *is firmly nonexpansive*,
(ii) $I-T$ *is firmly nonexpansive*,
(iii) $2T-I$ *is nonexpansive*,
(iv) $\|Tx-Ty\|^{2}\leq\langle x-y, Tx-Ty \rangle$, $\forall x, y \in H$,
(v) $0\leq\langle Tx-Ty, (I-T)x-(I-T)y\rangle$.

Recall $P_{C}: H\rightarrow C$ is an orthogonal projection, where *C* is a nonempty closed convex subset of *H*. Then to each point $x\in H$, the unique point $P_{C}x\in C$ satisfies the following property:

$$ \|x-P_{C}x\|=\inf_{y\in C}\|x-y\|=: d(x,C). $$

$P_{C}$ also has the following characteristics.

### Lemma 2.3

See [27]

*For a given* $x\in H$,

(i) $z=P_{C}x \Longleftrightarrow\langle x-z,z-y\rangle\geq0$, $\forall y\in C$,
(ii) $z=P_{C}x \Longleftrightarrow\|x-z\|^{2}\leq\|x-y\|^{2}-\|y-z\| ^{2}$, $\forall y\in C$,
(iii) $\langle P_{C}x-P_{C}y, x-y\rangle\geq\|P_{C}x-P_{C}y\|^{2}$, $\forall x,y\in H$.

### Lemma 2.4

See [28]

*Let* *f* *be given by* (1.4). *Then*

(i) *f* *is convex and differential*,
(ii) $\nabla f(x)=A^{*}(I-P_{Q})Ax$, $\forall x\in H$,
(iii) *f* *is w*-*lsc on* *H*,
(iv) ∇*f* *is* $\|A\|^{2}$-*Lipschitz*: $\|\nabla f(x)-\nabla f(y)\|\leq\|A\|^{2}\|x-y\|$, $\forall x,y\in H$.

### Lemma 2.5

See [29]

*Let* $\{a_{n}\}$ *be a sequence of nonnegative real numbers such that*

$$a_{n+1} \leq(1-\alpha_{n})a_{n} + \alpha_{n}\delta_{n}, \quad n \geq0, $$

*where* $\{\alpha_{n}\}_{n=0}^{\infty}$ *is a sequence in* $(0,1)$ *and* $\{ \delta_{n}\}_{n=0}^{\infty}$ *is a sequence in* $\mathbb{R}$ *such that*

(i) $\sum_{n=0}^{\infty}\alpha_{n} = \infty$,
(ii) $\limsup_{n\rightarrow\infty}\delta_{n} \leq0$ *or* $\sum_{n=0}^{\infty}\alpha_{n}|\delta_{n}| < \infty$.

*Then* $\lim_{n\rightarrow\infty}a_{n} = 0$.

### Lemma 2.6

See [30]

*Let* $\{\gamma_{n}\}_{n\in\mathbb{N}}$ *be a sequence of real numbers such that there exists a subsequence* $\{\gamma_{n_{i}}\}_{i\in \mathbb{N}}$ *of* $\{\gamma_{n}\}_{n\in\mathbb{N}}$ *such that* $\gamma _{n_{i}}<\gamma_{n_{i}+1}$ *for all* $i\in\mathbb{N}$. *Then there exists a nondecreasing sequence* $\{m_{k}\}_{k\in\mathbb{N}}$ *of* $\mathbb{N}$ *such that* $\lim_{k\rightarrow\infty}m_{k}=\infty$ *and the following properties are satisfied by all* (*sufficiently large*) *numbers* $k\in \mathbb{N}$:

$$ \gamma_{m_{k}}\leq\gamma_{m_{k}+1},\qquad \gamma_{k}\leq \gamma_{m_{k}+1}. $$

*In fact*, $m_{k}$ *is the largest number n in the set* $\{1,\ldots,k\}$ *such that the condition*

$$ \gamma_{n}\leq\gamma_{n+1} $$

*holds*.

## Main results

In this paper, we always assume that $f:H\rightarrow\mathbb{R}$ is a real-valued convex function, where $f(x)=\frac{1}{2}\|(I-P_{Q})Ax\|^{2}$, the gradient $\nabla f(x)=A^{*}(I-P_{Q})Ax$, *C* and *Q* are nonempty closed convex subsets of real Hilbert spaces $H_{1}$ and $H_{2}$, and $A:H_{1}\rightarrow H_{2}$ is a bounded linear operator.

### Algorithm 3.1

Choose an initial guess $x_{0}\in H$ arbitrarily. Assume that the *n*th iterate $x_{n}\in C$ has been constructed and $\nabla f(x_{n})\neq0$. Then we calculate the $(n+1)$th iterate $x_{n+1}$ via the formula

3.1 $$ x_{n+1}=P_{C}\bigl(x_{n}-\lambda_{n} \bigl(A^{*}(I-P_{Q})Ax_{n}+\beta_{n}x_{n} \bigr)\bigr), \quad\forall n\geq0, $$

where $\lambda_{n}$ is chosen as follows:

$$\lambda_{n}=\frac{\rho_{n}f(x_{n})}{\|\nabla f(x_{n})\|^{2}} $$

with $0<\rho_{n}<4$. If $\nabla f(x_{n})= 0$, then $x_{n+1}=x_{n}$ is a solution of SFP (1.1) and the iterative process stops. Otherwise, we set $n:=n+1$ and go to (3.1) to evaluate the next iterate $x_{n+2}$.

### Theorem 3.1

*Suppose that* $S\neq\emptyset$ *and the parameters* $\{\beta_{n}\}$ *and* $\{\rho_{n}\}$ *satisfy the following conditions*:

(i) $\{\beta_{n}\}\subset(0,1)$, $\lim_{n\rightarrow\infty}\beta _{n}=0$, $\sum_{n=1}^{\infty}\beta_{n} = \infty$,
(ii) $\varepsilon\leq\rho_{n}\leq4-\varepsilon$ *for some* $\varepsilon>0$ *small enough*.

*Then the sequence* $\{x_{n}\}$ *generated by Algorithm* 3.1 *converges strongly to* $z\in S$, *where* ${z=P_{S}(0)}$.

### Proof

Let $x^{*}\in S$. Since minimization is an exactly fixed point of its projection mapping, we have $x^{*}=P_{C}x^{*}$ and $Ax^{*}=P_{Q}Ax^{*}$.

By (3.1) and the nonexpansivity of $P_{C}$, we derive

3.2 $$ \begin{aligned}[b] \big\| x_{n+1}-x^{*}\big\| ^{2} ={}&\big\| P_{C}\bigl(x_{n}- \lambda_{n}\bigl(A^{*}(I-P_{Q})Ax_{n}+\beta _{n}x_{n}\bigr)\bigr)-P_{C}x^{*}\big\| ^{2} \\ \leq{}&\big\| (1-\lambda_{n}\beta_{n})x_{n}-\lambda _{n}A^{*}(I-P_{Q})Ax_{n}-x^{*}\big\| ^{2} \\ ={}&\bigg\| \lambda_{n}\beta_{n}\bigl(-x^{*}\bigr) +(1-\lambda_{n}\beta_{n}) \biggl(x_{n}- \frac{\lambda_{n}}{1-\lambda_{n}\beta _{n}}A^{*}(I-P_{Q})Ax_{n}-x^{*}\biggr) \bigg\| ^{2} \\ ={}&\lambda_{n}\beta_{n}\big\| x^{*}\big\| ^{2} +(1- \lambda_{n}\beta_{n})\bigg\| x_{n}-\frac{\lambda_{n}}{1-\lambda_{n}\beta _{n}}A^{*}(I-P_{Q})Ax_{n}-x^{*} \bigg\| ^{2} \\ & -\lambda_{n}\beta_{n}(1-\lambda_{n} \beta_{n})\bigg\| x_{n}-\frac{\lambda _{n}}{1-\lambda_{n}\beta_{n}}A^{*}(I-P_{Q})Ax_{n} \bigg\| ^{2} \\ \leq{}&\lambda_{n}\beta_{n}\big\| x^{*}\big\| ^{2} +(1-\lambda_{n}\beta_{n})\bigg\| x_{n}- \frac{\lambda_{n}}{1-\lambda _{n}\beta_{n}}A^{*}(I-P_{Q})Ax_{n}-x^{*}\bigg\| ^{2}. \end{aligned}$$

Since $P_{Q}$ is firmly nonexpansive, from Lemma 2.2, we deduce that $I-P_{Q}$ is also firmly nonexpansive. Hence, we have

3.3 $$ \begin{aligned}[b] \bigl\langle A^{*}(I-P_{Q})Ax_{n}, x_{n}-x^{*}\bigr\rangle &=\bigl\langle (I-P_{Q})Ax_{n}, Ax_{n}-Ax^{*} \bigr\rangle \\ &=\bigl\langle (I-P_{Q})Ax_{n}-(I-P_{Q})Ax^{*}, Ax_{n}-Ax^{*}\bigr\rangle \\ &\geq\big\| (I-P_{Q})Ax_{n}\big\| ^{2} \\ &=2f(x_{n}). \end{aligned}$$

Note that $\nabla f(x_{n})=A^{*}(I-P_{Q})Ax_{n}$. From (3.3), we obtain

3.4 $$\begin{aligned} &\bigg\| x_{n}-\frac{\lambda_{n}}{1-\lambda_{n}\beta _{n}}A^{*}(I-P_{Q})Ax_{n}-x^{*} \bigg\| ^{2} \\ &\quad=\big\| x_{n}-x^{*}\big\| ^{2}+\frac{\lambda_{n}^{2}}{(1-\lambda_{n}\beta_{n})^{2}}\big\| A^{*}(I-P_{Q})Ax_{n}\big\| ^{2} -\frac{2\lambda_{n}}{1-\lambda_{n}\beta_{n}}\bigl\langle A^{*}(I-P_{Q})Ax_{n}, x_{n}-x^{*}\bigr\rangle \\ &\quad=\big\| x_{n}-x^{*}\big\| ^{2}+\frac{\lambda_{n}^{2}}{(1-\lambda_{n}\beta_{n})^{2}}\big\| \nabla f(x_{n})\big\| ^{2}-\frac{2\lambda_{n}}{1-\lambda_{n}\beta_{n}}\bigl\langle \nabla f(x_{n}), x_{n}-x^{*} \bigr\rangle \\ &\quad\leq\big\| x_{n}-x^{*}\big\| ^{2}+\frac{\lambda_{n}^{2}}{(1-\lambda_{n}\beta_{n})^{2}}\big\| \nabla f(x_{n})\big\| ^{2}-\frac{4\lambda_{n}}{1-\lambda_{n}\beta _{n}}f(x_{n}) \\ &\quad=\big\| x_{n}-x^{*}\big\| ^{2}+\frac{1}{(1-\lambda_{n}\beta_{n})^{2}}\cdot \frac{\rho _{n}^{2}f(x_{n})^{2}}{\|\nabla f(x_{n})\|^{4}}\cdot\big\| \nabla f(x_{n})\big\| ^{2} \\ &\qquad{} -\frac{4\rho_{n}f(x_{n})}{(1-\lambda_{n}\beta_{n})\|\nabla f(x_{n})\| ^{2}}\cdot f(x_{n}) \\ &\quad=\big\| x_{n}-x^{*}\big\| ^{2}+\frac{\rho_{n}^{2}f(x_{n})^{2}}{(1-\lambda_{n}\beta _{n})^{2}\|\nabla f(x_{n})\|^{2}}-\frac{4\rho_{n}f(x_{n})^{2}}{(1-\lambda _{n}\beta_{n})\|\nabla f(x_{n})\|^{2}} \\ &\quad=\big\| x_{n}-x^{*}\big\| ^{2} -\rho_{n}\biggl(4- \frac{\rho_{n}}{1-\lambda_{n}\beta_{n}}\biggr)\cdot\frac {f(x_{n})^{2}}{(1-\lambda_{n}\beta_{n})\|\nabla f(x_{n})\|^{2}}. \end{aligned}$$

By condition (ii), without loss of generality, we assume that $(4-\frac{\rho_{n}}{1-\lambda_{n}\beta_{n}})>0$ for all $n\geq0 $. Thus from (3.2) and (3.4), we obtain

3.5 $$ \begin{aligned}[b] \big\| x_{n+1}-x^{*}\big\| ^{2} \leq{}&\lambda_{n} \beta_{n}\big\| x^{*}\big\| ^{2}+(1-\lambda_{n} \beta_{n}) \biggl(\big\| x_{n}-x^{*}\big\| ^{2} \\ &-\rho_{n}\biggl(4-\frac{\rho_{n}}{1-\lambda_{n}\beta_{n}}\biggr)\cdot\frac {f(x_{n})^{2}}{(1-\lambda_{n}\beta_{n})\|\nabla f(x_{n})\|^{2}} \biggr) \\ ={}&\lambda_{n}\beta_{n}\big\| x^{*}\big\| ^{2}+(1- \lambda_{n}\beta_{n})\big\| x_{n}-x^{*}\big\| ^{2} \\ & -\rho_{n}\biggl(4-\frac{\rho_{n}}{1-\lambda_{n}\beta_{n}}\biggr)\cdot\frac {f(x_{n})^{2}}{\|\nabla f(x_{n})\|^{2}} \\ \leq{}&\lambda_{n}\beta_{n}\big\| x^{*}\big\| ^{2}+(1- \lambda_{n}\beta_{n})\big\| x_{n}-x^{*}\big\| ^{2} \\ \leq{}&\max\bigl\{ \big\| x^{*}\big\| ^{2}, \big\| x_{n}-x^{*}\big\| ^{2} \bigr\} . \end{aligned}$$

Hence, $\{x_{n}\}$ is bounded.

Let $z=P_{S}0$. From (3.5), we deduce

3.6 $$ \begin{aligned}[b] 0 &\leq\rho_{n}\biggl(4-\frac{\rho_{n}}{1-\lambda_{n}\beta_{n}}\biggr)\frac {f(x_{n})^{2}}{\|\nabla f(x_{n})\|^{2}} \\ &\leq\lambda_{n}\beta_{n}\|z\|^{2}+(1- \lambda_{n}\beta_{n})\|x_{n}-z\| ^{2}- \|x_{n+1}-z\|^{2}. \end{aligned}$$

We consider the following two cases.

Case 1. One has $\|x_{n+1}-z\|\leq\|x_{n}-z\|$ for every $n\geq n_{0}$ large enough.

In this case, $\lim_{n\rightarrow\infty}\|x_{n}-z\|$ exists as finite and hence

3.7 $$ \lim_{n\rightarrow\infty}\bigl(\|x_{n+1}-z\|-\|x_{n}-z\|\bigr)=0. $$

This, together with (3.6), implies that

$$ \rho_{n}\biggl(4-\frac{\rho_{n}}{1-\lambda_{n}\beta_{n}}\biggr)\frac{f(x_{n})^{2}}{\| \nabla f(x_{n})\|^{2}} \rightarrow0. $$

Since $\liminf_{n\rightarrow\infty}\rho_{n}(4-\frac{\rho_{n}}{1-\lambda _{n}\beta_{n}})\geq\varepsilon_{0}$ (where $\varepsilon_{0}>0$ is a constant), we get

$$\frac{f(x_{n})^{2}}{\|\nabla f(x_{n})\|^{2}}\rightarrow0. $$

Noting that $\|\nabla f(x_{n})\|^{2}$ is bounded, we deduce immediately that

3.8 $$ \lim_{n\rightarrow\infty}f(x_{n})=0. $$

Next, we prove that

3.9 $$ \limsup_{n\rightarrow\infty}\langle-z, x_{n}-z\rangle\leq0. $$

Since $\{x_{n}\}$ is bounded, there exists a subsequence $\{x_{n_{i}}\} $ satisfying $x_{n_{i}}\rightharpoonup\hat{z}$ and

$$ \limsup_{n\rightarrow\infty}\langle-z, x_{n}-z\rangle=\lim _{i\rightarrow\infty}\langle -z, x_{n_{i}}-z\rangle. $$

By the lower semicontinuity of *f*, we get

$$ 0\leq f(\hat{z})\leq\liminf_{i\rightarrow\infty}f(x_{n_{i}})=\lim _{n\rightarrow\infty}f(x_{n})=0. $$

So

$$ f(\hat{z})=\frac{1}{2}\big\| (I-P_{Q})A\hat{z}\big\| ^{2}=0. $$

That is, *ẑ* is a minimizer of *f*, and $\hat{z}\in S$. Therefore

3.10 $$ \begin{aligned}[b] \limsup_{n\rightarrow\infty}\langle-z, x_{n}-z\rangle &=\lim _{i\rightarrow\infty}\langle -z, x_{n_{i}}-z\rangle \\ &=\langle-z, \hat{z}-z\rangle \\ &\leq 0.\end{aligned} $$

Then we have

$$\begin{aligned} \|x_{n+1}-z\|^{2} ={}&\big\| P_{C}\bigl(x_{n}- \lambda_{n}A^{*}(I-P_{Q})Ax_{n}-\lambda_{n} \beta _{n}x_{n}\bigr)-P_{C}z\big\| ^{2} \\ \leq{}&\big\| (1-\lambda_{n}\beta_{n})x_{n}- \lambda_{n}A^{*}(I-P_{Q})Ax_{n}-z\big\| ^{2} \\ ={}&\bigg\| \lambda_{n}\beta_{n}(-z) +(1-\lambda_{n}\beta_{n}) \biggl(x_{n}- \frac{\lambda_{n}}{1-\lambda_{n}\beta _{n}}A^{*}(I-P_{Q})Ax_{n}-z\biggr)\bigg\| ^{2} \\ ={}&(\lambda_{n}\beta_{n})^{2}\|z\|^{2} \\ &+(1-\lambda_{n}\beta_{n})^{2}\bigg\| x_{n}- \frac{\lambda_{n}}{1-\lambda _{n}\beta_{n}}A^{*}(I-P_{Q})Ax_{n}-z\bigg\| ^{2} \\ &+2(1-\lambda_{n}\beta_{n})\lambda_{n} \beta_{n}\biggl\langle x_{n}-\frac {\lambda_{n}}{1-\lambda_{n}\beta_{n}}A^{*}(I-P_{Q})Ax_{n}-z,-z \biggr\rangle \\ \leq{}&(1-\lambda_{n}\beta_{n})^{2} \|x_{n}-z\|^{2}+(\lambda_{n}\beta_{n})^{2} \| z\|^{2} \\ & +2(1-\lambda_{n}\beta_{n})\lambda_{n} \beta_{n}\langle x_{n}-z, -z\rangle+2\lambda_{n}^{2} \beta_{n}\bigl\langle \nabla f(x_{n}), z\bigr\rangle \\ \leq{}&(1-\lambda_{n}\beta_{n})\|x_{n}-z \|^{2} \\ &+\lambda_{n}\beta_{n}\bigl(\lambda _{n}\beta_{n}\|z\|^{2}+2(1-\lambda_{n}\beta_{n})\langle x_{n}-z, -z \rangle+2\lambda_{n}\big\| \nabla f(x_{n})\big\| \cdot\|z\|\bigr). \end{aligned}$$

Note that $\|\nabla f(x_{n})\|^{2}$ is bounded, and that $\lambda_{n}\| \nabla f(x_{n})\|=\frac{\rho_{n}f(x_{n})}{\|\nabla f(x_{n})\|^{2}}\cdot\| \nabla f(x_{n})\|$. Thus $\lambda_{n}\|\nabla f(x_{n})\|\rightarrow0$ by (3.8). From Lemma 2.5, we deduce that

$$x_{n}\rightarrow z. $$

Case 2. There exists a subsequence $\{\|x_{n_{j}}-z\|\}$ of $\{\| x_{n}-z\|\}$ such that

$$\|x_{n_{j}}-z\|< \|x_{n_{j}+1}-z\| \quad\text{for all }j\geq1. $$

By Lemma 2.6, there exists a strictly nondecreasing sequence $\{m_{k}\} $ of positive integers such that $\lim_{k\rightarrow\infty}m_{k}=+\infty $ and the following properties are satisfied by all numbers $k\in \mathbb{N}$:

3.11 $$ \|x_{m_{k}}-z\|\leq\|x_{m_{k}+1}-z\|,\qquad \|x_{k}-z\|\leq \|x_{m_{k}+1}-z\|. $$

We have

$$\begin{aligned} \|x_{n+1}-z\| ={}&\big\| P_{C}\bigl(x_{n}- \lambda_{n}A^{*}(I-P_{Q})Ax_{n}-\lambda_{n} \beta _{n}x_{n}\bigr)-P_{C}z\big\| \\ \leq{}&\big\| (1-\lambda_{n}\beta_{n})x_{n}- \lambda_{n}A^{*}(I-P_{Q})Ax_{n}-z\big\| \\ ={}&\bigg\| \lambda_{n}\beta_{n}(-z) +(1-\lambda_{n}\beta_{n}) \biggl(x_{n}- \frac{\lambda_{n}}{1-\lambda_{n}\beta _{n}}A^{*}(I-P_{Q})Ax_{n}-z\biggr)\bigg\| \\ \leq{}&\lambda_{n}\beta_{n}\big\| (-z)\big\| +(1-\lambda_{n} \beta_{n})\bigg\| x_{n}-\frac {\lambda_{n}}{1-\lambda_{n}\beta_{n}}A^{*}(I-P_{Q})Ax_{n}-z \bigg\| \\ \leq{}&\lambda_{n}\beta_{n}\|z\|+(1-\lambda_{n} \beta_{n})\|x_{n}-z\|. \end{aligned}$$

Consequently,

$$\begin{aligned} 0 &\leq\lim_{k\rightarrow\infty}\bigl(\|x_{m_{k}+1}-z\|-\|x_{m_{k}}-z \|\bigr) \\ &\leq\limsup_{n\rightarrow\infty}\bigl(\|x_{n+1}-z\|-\|x_{n}-z \|\bigr) \\ &\leq\limsup_{n\rightarrow\infty}\bigl(\lambda_{n} \beta_{n}\|z\|+(1-\lambda _{n}\beta_{n}) \|x_{n}-z\|-\|x_{n}-z\|\bigr) \\ &=\limsup_{n\rightarrow\infty}\lambda_{n}\beta_{n}\bigl(\|z \|-\|x_{n}-z\|\bigr) \\ &=0. \end{aligned}$$

Hence,

3.12 $$ \lim_{k\rightarrow\infty}\bigl(\|x_{m_{k}+1}-z\|-\|x_{m_{k}}-z\|\bigr)=0. $$

By a similar argument to that of Case 1, we prove that

3.13 $$\begin{aligned}& \limsup_{k\rightarrow\infty}\langle-z, x_{m_{k}}-z \rangle\leq0, \\& \|x_{m_{k}+1}-z\|^{2}\leq(1-\lambda_{m_{k}} \beta_{m_{k}})\|x_{m_{k}}-z\| ^{2}+\lambda_{m_{k}} \beta_{m_{k}}\sigma_{m_{k}}, \end{aligned}$$

where

$$\sigma_{m_{k}}=\lambda_{m_{k}}\beta_{m_{k}}\|z \|^{2}+2(1-\lambda _{m_{k}}\beta_{m_{k}})\langle x_{m_{k}}-z, -z\rangle+2\lambda_{m_{k}}\big\| \nabla f(x_{m_{k}})\big\| \cdot\|z\|. $$

In particular, from (3.13), we get

3.14 $$ \lambda_{m_{k}}\beta_{m_{k}}\|x_{m_{k}}-z\|^{2} \leq\|x_{m_{k}}-z\|^{2}-\| x_{m_{k}+1}-z\|^{2}+ \lambda_{m_{k}}\beta_{m_{k}}\sigma_{m_{k}}. $$

Since $\|x_{m_{k}}-z\|\leq\|x_{m_{k}+1}-z\|$, we deduce that

$$ \|x_{m_{k}}-z\|^{2}-\|x_{m_{k}+1}-z\|^{2}\leq0. $$

Then, from (3.14), we have

$$ \lambda_{m_{k}}\beta_{m_{k}}\|x_{m_{k}}-z\|^{2} \leq\lambda_{m_{k}}\beta _{m_{k}}\sigma_{m_{k}}. $$

Then

3.15 $$ \limsup_{k\rightarrow\infty}\|x_{m_{k}}-z\|^{2}\leq\limsup _{k\rightarrow \infty}\sigma_{m_{k}}\leq0. $$

Then, from (3.12), we deduce that

3.16 $$ \limsup_{k\rightarrow\infty}\|x_{m_{k}+1}-z\|=0. $$

Thus, from (3.11) and (3.16), we conclude that

$$ \limsup_{k\rightarrow\infty}\|x_{k}-z\|\leq\limsup _{k\rightarrow\infty }\|x_{m_{k}+1}-z\|=0. $$

Therefore, $x_{n}\rightarrow z$. This completes the proof. □

## Conclusion

Recently, the SFP has been studied extensively by many authors. However, some algorithms need to compute $\|A\|$, and this is not an easy thing to work out. Others do not need to compute $\|A\|$, but the algorithms always have weak convergence. If we want to obtain strong convergence theorems, the algorithms are complex and difficult to calculate. We try to get over the drawbacks. In this article, we use the regularized CQ algorithm without computing $\|A\|$ to find the minimum-norm solution of the SFP, where $\lambda_{n}=\frac{\rho _{n}f(x_{n})}{\|\nabla f(x_{n})\|^{2}}$, $0<\rho_{n}<4$. Then, under suitable conditions, the explicit strong convergence theorem is obtained.

## References

[1] Censor Y, Elfving T (1994). A multiprojection algorithm using Bregman projections in a product space. Numer. Algorithms.

[2] Censor Y, Bortfeld T, Martin B, Trofimov A (2003). A unified approach for inversion problems in intensity-modulated radiation therapy. Phys. Med. Biol..

[3] Censor Y, Elfving T, Kopf N, Bortfeld T (2005). The multiple-sets split feasibility problem and its applications for inverse problem. Inverse Probl..

[4] López G, Martín-Márquez V, Xu HK (2009). Perturbation techniques for nonexpansive mappings with applications. Nonlinear Anal., Real World Appl..

[5] López G, Martín-Márquez V, Xu HK, Censor Y, Jiang M, Wang G (2010). Iterative algorithms for the multiple-sets split feasibility problem. Biomedical Mathematics: Promising Directions in Imaging, Therapy Planning and Inverse Problems.

[6] Xu HK (2010). Iterative methods for the split feasibility problem in infinite-dimensional Hilbert space. Inverse Probl..

[7] Dang Y, Gao Y (2011). The strong convergence of a KM-CQ-like algorithm for a split feasibility problem. Inverse Probl..

[8] Qu B, Xiu N (2005). A note on the CQ algorithm for the split feasibility problem. Inverse Probl..

[9] Wang F, Xu HK (2010). Approximating curve and strong convergence of the CQ algorithm for the split feasibility problem. J. Inequal. Appl..

[10] Xu HK (2006). A variable Krasnosel’skii-Mann algorithm and the multiple-set split feasibility problem. Inverse Probl..

[11] Yang Q (2004). The relaxed CQ algorithm for solving the split feasibility problem. Inverse Probl..

[12] Byrne C (2002). Iterative oblique projection onto convex sets and the split feasibility problem. Inverse Probl..

[13] Byrne C (2004). A unified treatment of some iterative algorithms in signal processing and image reconstruction. Inverse Probl..

[14] Yao YH, Wu JG, Liou YC (2012). Regularized methods for the split feasibility problem. Abstr. Appl. Anal..

[15] Yao YH, Liou YC, Yao JC (2017). Iterative algorithms for the split variational inequality and fixed point problems under nonlinear transformations. J. Nonlinear Sci. Appl..

[16] Yao YH, Agarwal RP, Postolache M, Liou YC (2014). Algorithms with strong convergence for the split common solution of the feasibility problem and fixed point problem. Fixed Point Theory Appl..

[17] Latif A, Qin X (2017). A regularization algorithm for a splitting feasibility problem in Hilbert spaces. J. Nonlinear Sci. Appl..

[18] Chang SS, Wang L, Zhao YH (2017). On a class of split equality fixed point problems in Hilbert spaces. J. Nonlinear Var. Anal..

[19] Tang JF, Chang SS, Dong J (2017). Split equality fixed point problem for two quasi-asymptotically pseudocontractive mappings. J. Nonlinear Funct. Anal..

[20] Shi LY, Ansari QH, Wen CF, Yao JC (2017). Incremental gradient projection algorithm for constrained composite minimization problems. J. Nonlinear Var. Anal..

[21] Xu HK (2011). Averaged mappings and the gradient-projection algorithm. J. Optim. Theory Appl..

[22] Yang Q (2005). On variable-step relaxed projection algorithm for variational inequalities. J. Math. Anal. Appl..

[23] López G, Martín-Márquez V, Wang FH, Xu HK (2012). Solving the split feasibility problem without prior knowledge of matrix norms. Inverse Probl..

[24] Yao YH, Postolache M, Liou YC (2013). Strong convergence of a self-adaptive method for the split feasibility problem. Fixed Point Theory Appl..

[25] Bauschke HH, Combettes PL (2011). Convex Analysis and Monotone Operator Theory in Hilbert Spaces.

[26] Goebel K, Kirk WA (1990). Topics on Metric Fixed Point Theory.

[27] Takahashi W (2000). Nonlinear Functional Analysis.

[28] Aubin JP (1993). Optima and Equilibria: An Introduction to Nonlinear Analysis.

[29] Xu HK (2004). Viscosity approximation methods for nonexpansive mappings. J. Math. Anal. Appl..

[30] Maingé PE (2008). Strong convergence of projected subgradient methods for non-smooth and non-strictly convex minimization. Set-Valued Anal..

